# Author Correction: Ocean heat forced West Antarctic Ice Sheet retreat after the Last Glacial Maximum

**DOI:** 10.1038/s41467-026-72192-3

**Published:** 2026-04-17

**Authors:** Elaine M. Mawbey, James A. Smith, Claus-Dieter Hillenbrand, Katharine R. Hendry, Erin L. McClymont, Mervyn J. Greaves, Johann P. Klages, Gerhard Kuhn, Svetlana Radionovskaya, Charlotte L. Spencer-Jones, Robert D. Larter, Julia S. Wellner, Pierre Dutrieux

**Affiliations:** 1 British Antarctic Survey, High Cross, Madingley Road, Cambridge, UK; 2 Department of Geography, Durham University, Lower Mountjoy, South Road, Durham, UK; 3 The Godwin Laboratory for Palaeoclimate Research, Department of Earth Sciences, University of Cambridge, Downing Street, Cambridge, UK; 4 Alfred-Wegener-Institut Helmholtz-Zentrum für Polar- und Meeresforschung, Am Alten Hafen 26, Bremerhaven, Germany; 5 Department of Earth and Atmospheric Sciences, University of Houston, Houston, TX USA

**Keywords:** Cryospheric science, Palaeoclimate

Correction to: *Nature Communications* 10.1038/s41467-026-68949-5, published online 6 February 2026

In the version of this article initially published, there was an error in Fig. 1 and associated Supplementary Data where a core ID was incorrectly labelled as PS75/191, when it should have been PS75/192, as is now amended in the HTML and PDF versions of the article.

