# Supplementary material for: Ocean heat forced West Antarctic Ice Sheet retreat after the Last Glacial Maximum
Source: Nat Commun. 2026 Feb 6;17:2079. doi: 10.1038/s41467-026-68949-5 (PMC12953795; doi:10.1038/s41467-026-68949-5)
Supplement: Supplementary file 1 — Supplementary Information [file 41467_2026_68949_MOESM1_ESM.pdf]

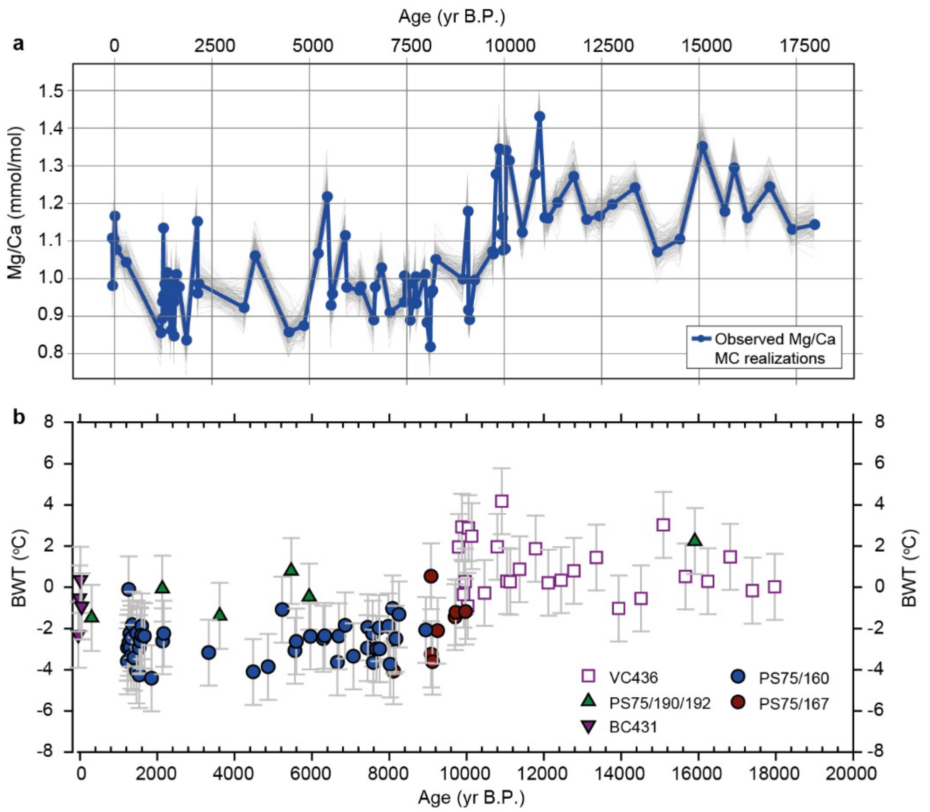

Supplementary Figure 1 I a. 2 standard deviation error of 10000 Monte Carlo realisations (grey shading) for Mn corrected Mg/Ca data (blue line). **b.** Bottom water temperatures (BWT) calculated from Mn corrected Mg/Ca of shells of the benthic foraminifera species *Trifarina angulosa* versus age using the calibration of Mawbey et al.[26]. Error bars represent ± 2 standard deviations (s.d.) from panel a (see Methods).

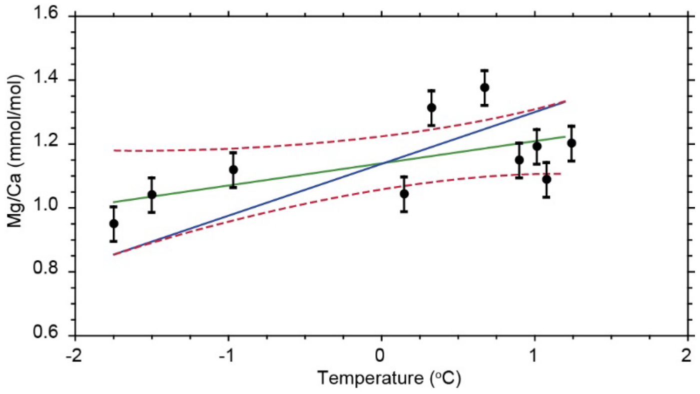

Supplementary Figure 2 I Mg/Ca-temperature calibration model for *T. angulosa* shells in seafloor surface sediment samples from various Antarctic shelf sectors (green line; BWT(°C) = (Mg/Ca−1.14 ± 0.035)/0.069 ± 0.033)[26] and calibration adjusted to reflect modern temperature ranges (blue line; BWT(°C) = (Mg/Ca−1.14 ± 0.035)/0.069 ± 0.033*0.3), equating to a correction factor of 0.3. Red dashed line represents the 95% confidence interval of the calibration (see Methods), while the error bars are based on a repeatability study on *Uvigerina* spp. at the same analytical facility[87].

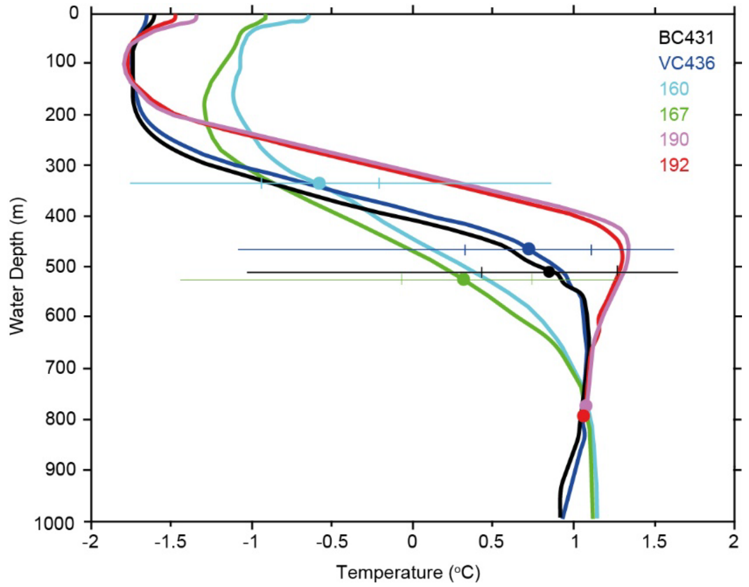

Supplementary Figure 3 I Average seawater temperature profiles for studied core sites versus water depth. Average temperature profiles are derived from CTD casts within a 60 km radius of each core site, throughout the entire measurement record (1994-present). The corresponding horizontal lines represent the seasonal deep-water temperature variability at the water depth of each site (filled circle), with maximum and minimum temperatures denoted by vertical ticks. Mean temperatures are as follows: BC431 = 0.852°C±0.421°C, VC436 = 0.726°C±0.391°C, PS75/160 = -0.573°C±0.370°C, PS75/167 = 0.342°C±0.403°C, PS75/190 =1.078°C±0.015 °C, PS75/192 = 1.074°C±0°C.

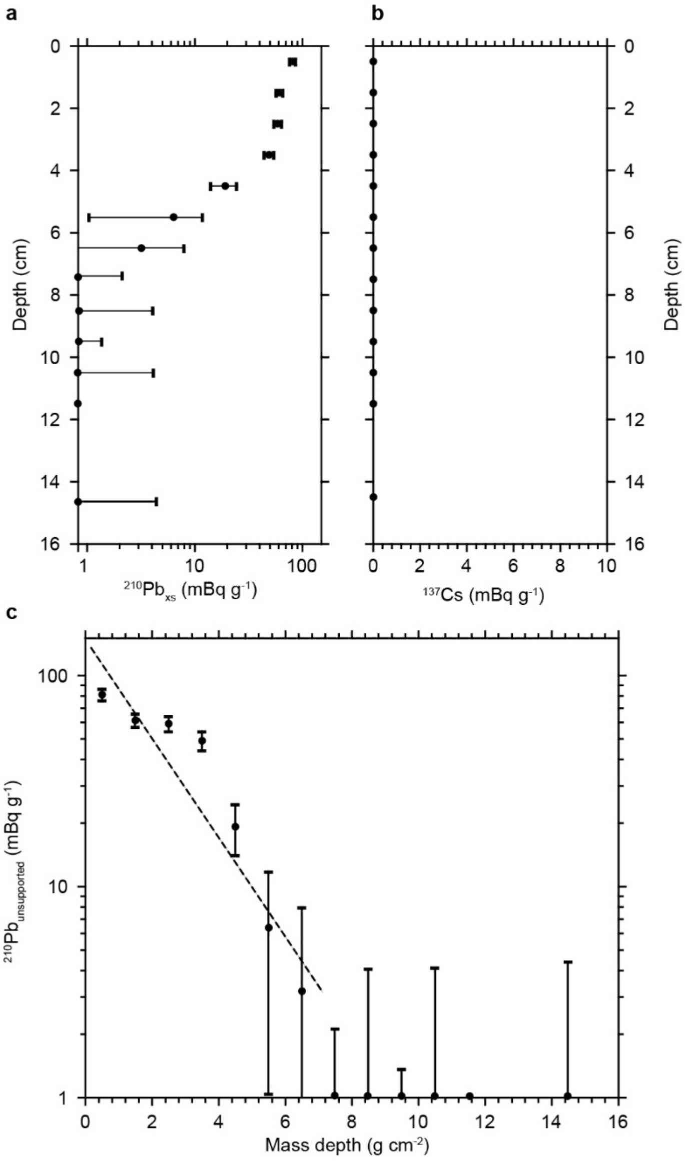

Supplementary Figure 4 I Down-core concentrations of ^{210}Pb and ^{137}Cs in core BC431. a. Concentration of unsupported ^{210}Pb ($^{210}Pb_{xs}$) for BC431. Error bars denote ±1 standard deviation (s.d). The $^{210}Pb_{xs}$ concentration is 81 mBq/g at the sediment surface and declines exponentially with core depth in the upper 6.5 cm. The ^{210}Pb activity is at, or below, the detection limit below 6.5 cm core depth. **b.** ^{137}Cs activity in BC431. The ^{137}Cs activity is at or below the detection limit throughout the core. **c.** Constant rate of supply (CRS) modelling of $^{210}Pb_{xs}$ profile from BC431. CRS modelling was conducted on the sediments from BC431 following Appleby[74]. The dashed line marks the regression used to calculate the ^{210}Pb concentration below 6 cm core depth (Supplementary Figure 5).

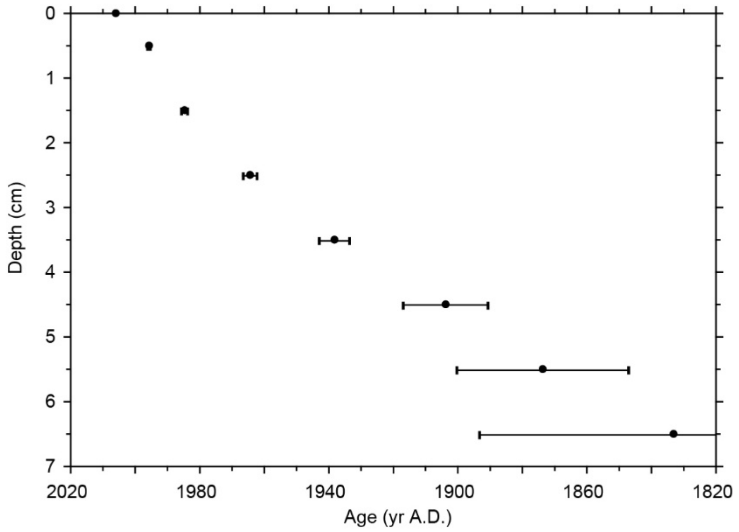

Supplementary Figure 5 I Age–depth model for core BC431. Ages are based on CRS modelling of the down-core $^{210}Pb_{xs}$ profile in the uppermost part of the core (Supplementary Figure 4c). Error bars denote ±1 s.d. of the calculated ages.

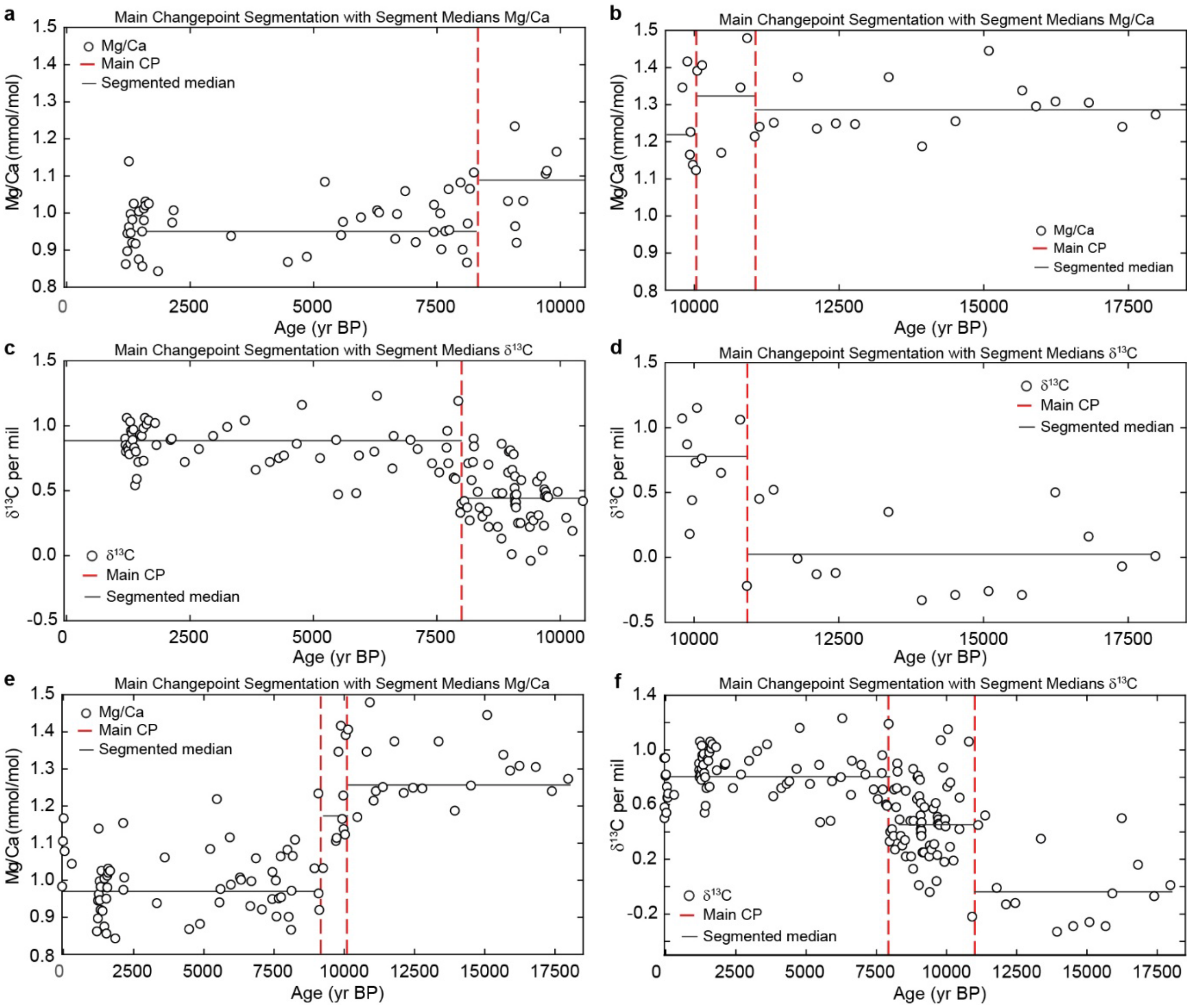

Supplementary Fig. 6 | Change-point (CP) analysis of Mg/Ca and δ^{13}C records. **a.** Inner-shelf Mg/Ca (CP at 8248 yr BP). **b.** Outer-shelf Mg/Ca with CPs at 11043 yr BP (warming) and 10050 yr BP (cooling). **c**. Inner shelf δ^{13}C (CP at 7977 yr BP). **d**. Outer-shelf δ^{13}C with a CP at 10911 yr BP. e-f. Composite Mg/Ca records showing CPs at 10135 and 9113 yr BP, and δ^{13}C CPs at 10911 and 7938 yr BP. Vertical dashed lines denote the CP, while horizontal black lines denote the segmented means.

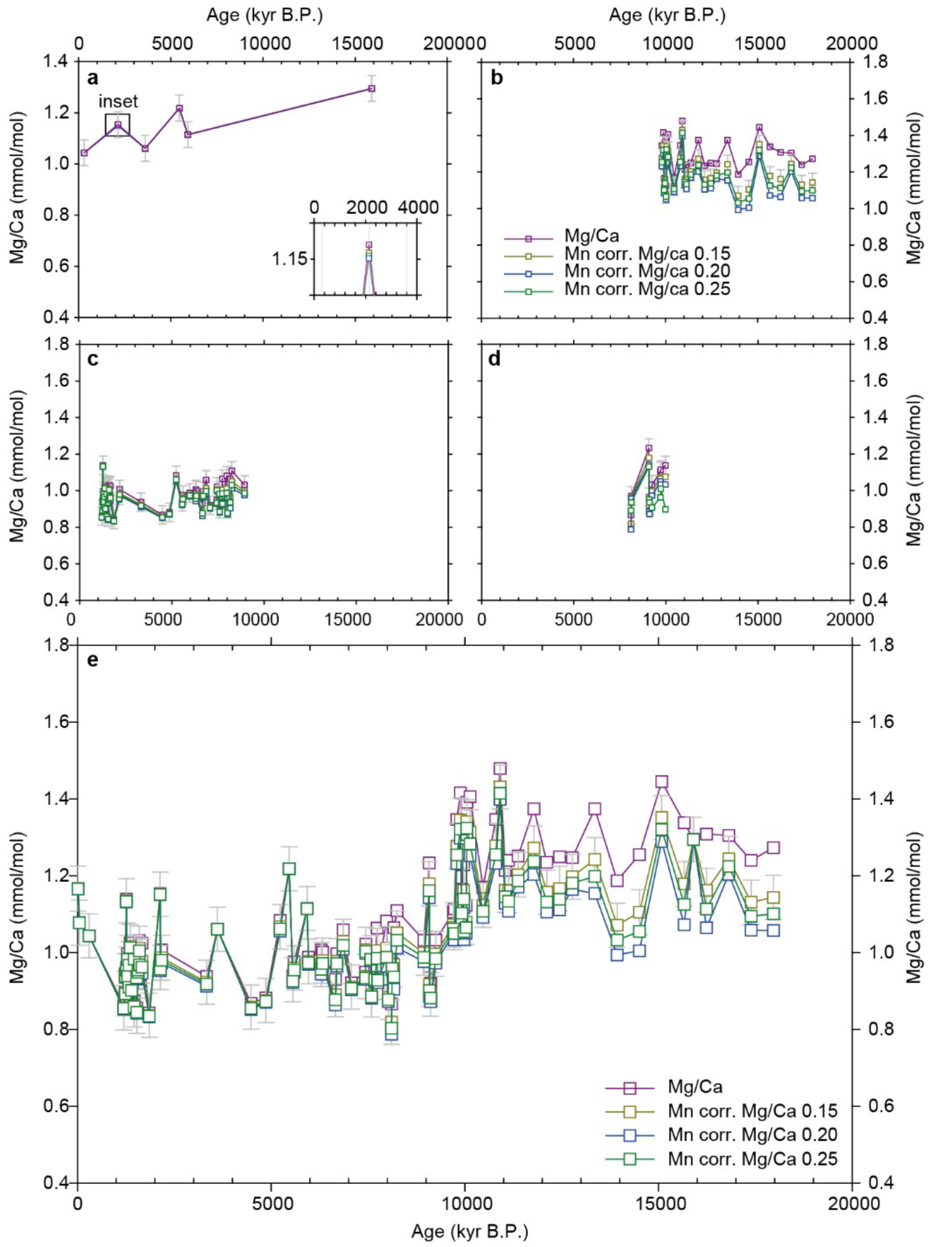

Supplementary Figure 7 I Mg/Ca ratios of benthic foraminifer shells in the Amundsen Sea. Mg/Ca (purple line) with adjustment for a potential diagenetic Mg contribution by assuming a magnesium/manganese (Mg/Mn) ratio of 0.15 ± 0.05 mol/mol in the diagenetic coating (yellow line) following Hillenbrand et al.[21]. Also shown are potential diagenetic Mg contributions assuming Mg/Mn ratios in diagenetic coatings of 0.20 (blue line) and 0.25 mol/mol (green line) following Hasenfratz et al.[82] **a.** PS75/190/192. **b.** VC436. **c.** PS75/160. **d.** PS75/167. **e.** Composite dataset. Error bars represent 2 standard deviations (s.d.).

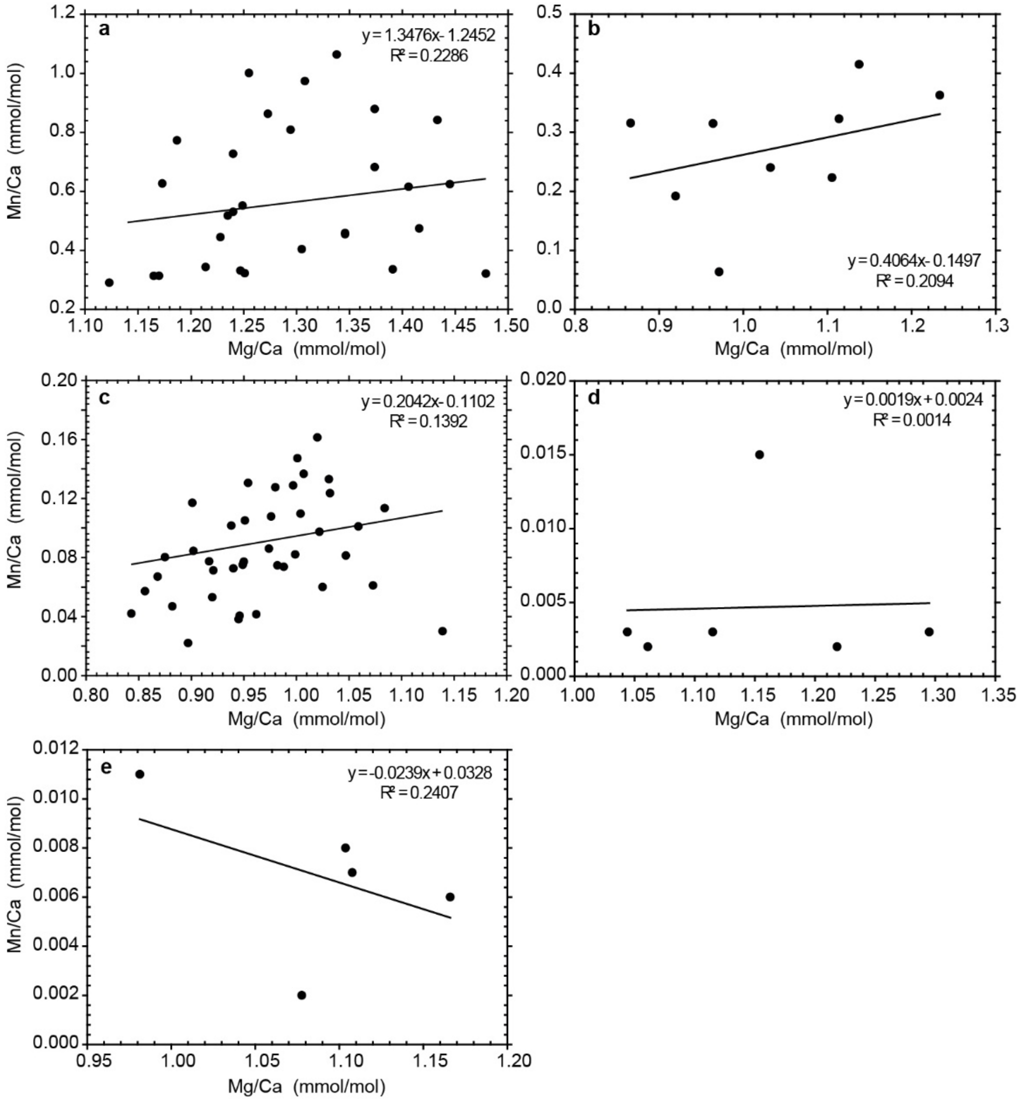

Supplementary Figure 8 I Mn/Ca vs Mg/Ca cross plots. a VC436. b. PS75/167, c. PS75/160. d. PS75/190/192. e. BC431.

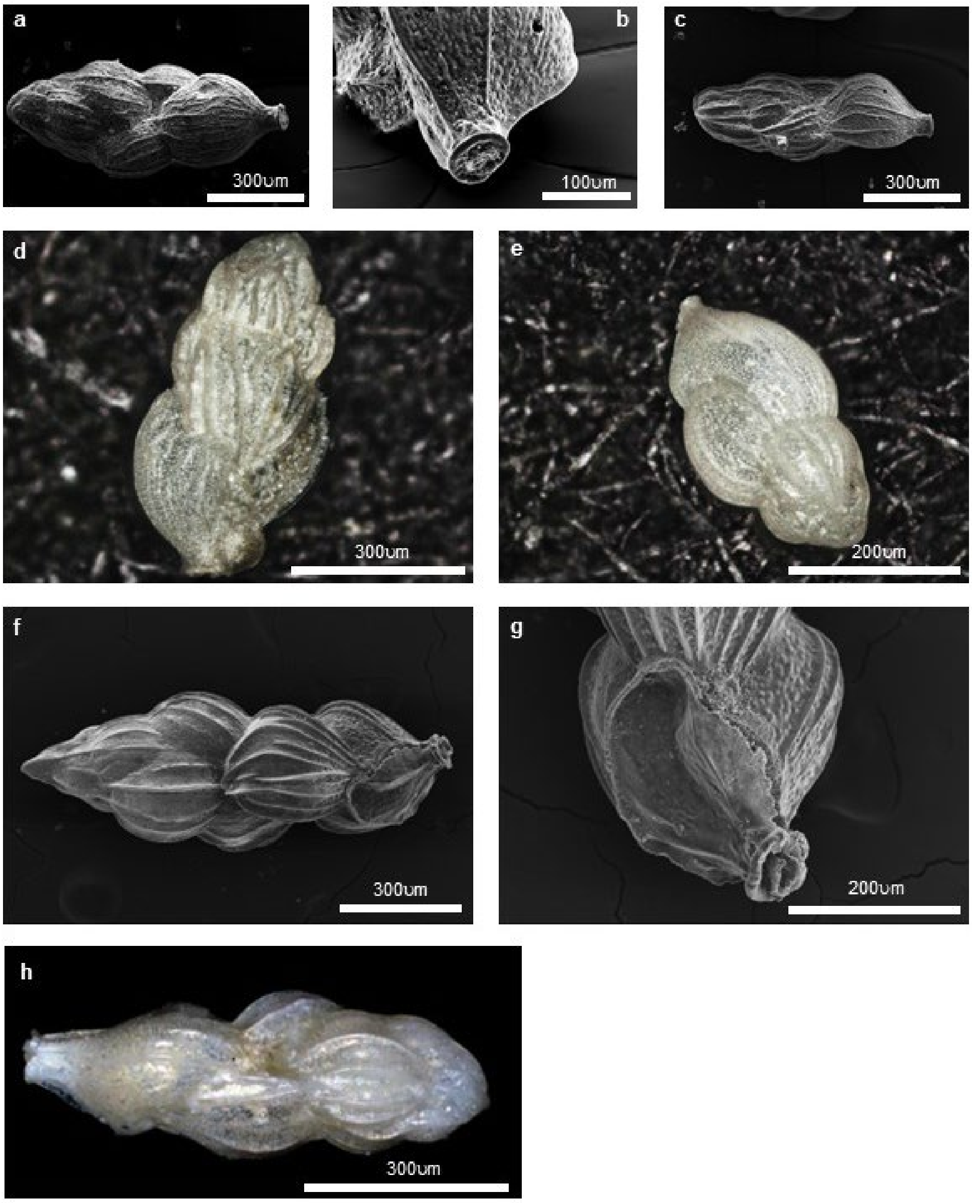

Supplementary Figure 9 | Representative SEM and light microscope images of benthic foraminifer *Trifarina angulosa* shells. **a-c.** BC431, 3 cm. **d-e.** PS75/190, 9 cm. **f-g.** PS75/160, 21cm. **h.** BC431, 1 cm.
