# Supplementary material for: Ocean heat forced West Antarctic Ice Sheet retreat after the Last Glacial Maximum
Source: Nat Commun. 2026 Feb 6;17:2079. doi: 10.1038/s41467-026-68949-5 (PMC12953795; doi:10.1038/s41467-026-68949-5)
Supplement: Supplementary file 2 — Description of Additional Supplementary Files [file 41467_2026_68949_MOESM2_ESM.pdf]

Description of Additional Supplementary Files

File name: Supplementary Data 1

Description: Mg/Ca data values of benthic foraminifera *T. angulosa* together with calculated and scaled bottom water temperature (BWT) (Methods). ±2 standard deviation (s.d.) error is derived from 10000 Monte Carlo realisations (Methods).

File name: Supplementary Data 2

Description: $\delta^{13}C$ data values of benthic foraminifera *T. angulosa*. $\delta^{13}C$ data from PS75-160-1 and PS75-167-1 is from Hillenbrand et al[21] and is also available here https://doi.pangaea.de/10.1594/PANGAEA.875733.

File name: Supplementary Data 3

Description: AMS ^{14}C data for cores VC436 and BC435 from the same location. Previously published ages are from Smith et al.[25] and have been recalibrated using Marine20.

File name: Supplementary Data 4

Description: ^{210}Pb chronology for BC431. Ages are based on constant rate of supply (CRS) modelling of the down-core $^{210}Pb_{xs}$ profile in the uppermost part of the core (Supplementary Figure 4c). Error bars denote ±1 standard deviation (s.d.) of the calculated ages.
